# Supplementary material for: Clinical Application of the Association between Genetic Alteration and Intraoperative Fluorescence Activity of 5-Aminolevulinic Acid during the Resection of Brain Metastasis of Lung Adenocarcinoma
Source: Cancers (Basel). 2023 Dec 23;16(1):88. doi: 10.3390/cancers16010088 (PMC10778171; doi:10.3390/cancers16010088)
Supplement: Supplementary file 1 [file cancers-16-00088-s001.zip › Supplementary Figure S1.pdf]

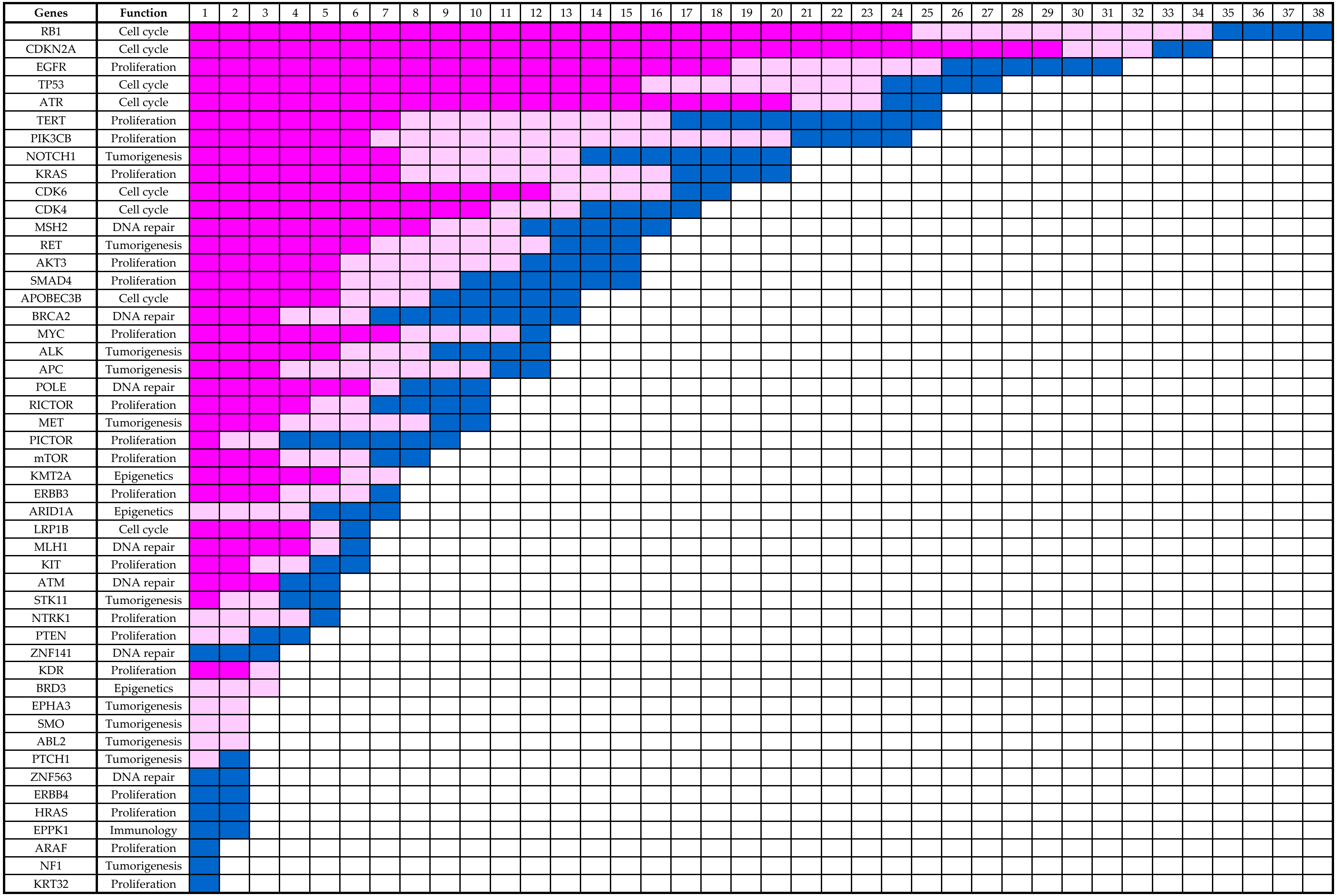

Supplementary Figure S1. Forty-nine genetic alterations in brain metastasis samples which were analyzed by Next-Generation Sequencing and fluorescent activity of 5-ALA.
