# Supplementary material for: Clinical Application of the Association between Genetic Alteration and Intraoperative Fluorescence Activity of 5-Aminolevulinic Acid during the Resection of Brain Metastasis of Lung Adenocarcinoma
Source: Cancers (Basel). 2023 Dec 23;16(1):88. doi: 10.3390/cancers16010088 (PMC10778171; doi:10.3390/cancers16010088)
Supplement: Supplementary file 1 [file cancers-16-00088-s001.zip › Supplementary Table S1.pdf]

Supplementary Table S1. List of Genes of ONCOaccuPanel for Next Generation Sequencing.

**Partial Exon and Hot Spot (98 genes)**														
A1BG	ABCC5	ACVR2A	ADAMTS18	ADNP	AKAP7	AP1S1	ARV1	ASH1L	BAX	BTK	CASD1	CBL	CBX4	CCDC73
CD3G	CDH26	CEBPZ	CENPV	CIC	CKAP2	CLOCK	COBLL1	CPEB2	CRIPAK	DLC1	DNAH12	DOCK3	DPAGT1	DYNC1I2
EBPL	EPPK1	FBXL3	FGFBP1	FMN2	FRG2B	FXR1	GRIN3B	GTPBP2	IFITM1	IFITM3	IMPA1	INO80E	IRS1	KCTD16
KLF4	KNSTRN	KRT32	LIPT1	MADCAM1	MAX	MFSD14A	MFSD4B	MVK	MYO1A	NFE2L2	NIPA2	NNAT	NOS3	NOTCH2NL
NUDT7	OR4M2	PABPC1	PCBP1	PCDHB16	PCMTD1	PPP2R1A	PREX2	PRIM2	RAC1	RASA4	RBBP8	RGS12	RHOA	RUFY2
SEC63	SF3B1	SLC23A2	SPRR3	SSTR4	STAMBPL1	STAT3	STAU2	SULT6B1	SYNJ2	TAS2R19	TAS2R31	TCF7L2	TEAD2	TMEM60
TMPRSS13	TPSD1	U2AF1	WDR55	WDR87	ZFP37	ZNF141	ZNF563							
**Entire Coding Gene Exons (225 genes)**														
ALK	BRCA1	BRCA2	BRAF	EGFR	ERBB2	IDH1	IDH2	KRAS	KIT	MYC	MYCN	NRAS	PDGFRA	NTRK1
RET	ROS1	ABL1	ABL2	ABRAXAS1	AKT1	AKT2	AKT3	APC	AR	ARAF	ARID1A	ARID1B	ARID2	ASXL1
ATM	ATR	ATRX	AURKA	AURKB	AURKC	AXIN1	AXL	BAP1	BARD1	BRD2	BRD3	BRD4	BRIP1	CBFB
CCND1	CCND2	CCND3	CCNE1	CD274	CDH1	CDK12	CDK4	CDK6	CDKN1A	CDKN1B	CDKN2A	CDKN2B	CDKN2C	CEBPA
CHEK2	CREBBP	CSF1R	CTNNB1	DDR1	DDR2	DDX3X	DNMT3A	DOT1L	DPYD	EPHA3	EPHB4	ERBB3	ERBB4	ERCC2
ERCC4	ERG	ERRFI1	ESR1	ETV1	ETV4	ETV5	ETV6	EWSR1	EZH2	FANCA	FANCB	FANCC	FANCD2	FANCE
FANCF	FANCG	FANCI	FANCL	FANCM	FBXW7	FGF19	FGF4	FGFR1	FGFR2	FGFR3	FGFR4	FLCN	FLT1	FLT3
FLT4	FOXL2	FUBP	GATA2	GEN1	GNA11	GNAQ	GNAS	H3F3A	HDAC9	HGF	HLA-A	HLA-B	HLA-C	HLA-DRB1
HNF1A	HRAS	IGF1R	IGF2	JAK1	JAK2	JAK3	KDR	KMT2A	LRP1B	LTK	MAP2K1	MAP2K2	MAP2K4	MAP3K1
MAP3K4	MAPK1	MAPK3	MAPK8	MCL1	MDM2	MDM4	MED12	MEN1	MET	MITF	MLH1	MPL	MRE11	MSH2
MSH6	MTAP	MTOR	NBN	NF1	NF2	NFKBIA	NKX2-1	NOTCH1	NOTCH2	NOTCH3	NOTCH4	NTRK2	NTRK3	NUTM1
PBRM1	PDGFB	PDGFRB	PIK3CA	PIK3CB	PIK3CD	PIK3R1	PIK3R2	PMS2	POLE	PPARG	PTCH1	PTCH2	PTEN	PTPN11
PALB2	PARP1	RAD50	RAD51	RAD51C	RAD51D	RAF1	RARA	RB1	RICTOR	RNF43	RSPO1	RSPO2	RUNX1	SDHA
SDHB	SDHC	SDHD	SETD2	SLX4	SMAD2	SMAD4	SMARCA4	SMARCB1	SMO	SOX2	SOX9	SPOP	SRC	STK11
SYK	TERT	TET2	TMPRSS2	TOP1	TOP2A	TP53	TSC1	TSC2	UBE2T	VHL	WT1	XPO1	XRCC2	ZNRF3
